# Metformin and trametinib have synergistic effects on cell viability and tumor growth in *NRAS* mutant cancer

**DOI:** 10.18632/oncotarget.2824

**Published:** 2014-11-25

**Authors:** Igor Vujic, Martina Sanlorenzo, Christian Posch, Rosaura Esteve-Puig, Adam J. Yen, Andrew Kwong, Aaron Tsumura, Ryan Murphy, Klemens Rappersberger, Susana Ortiz-Urda

**Affiliations:** ^1^ University of California, San Francisco, Department of Dermatology, Mt. Zion Cancer Research Center, San Francisco, CA, USA; ^2^ Rudolfstiftung Hospital, Academic Teaching Hospital, Department of Dermatology, Juchgasse, Vienna, Austria; ^3^ Department of Medical Sciences, Section of Dermatology, University of Turin, Italy

**Keywords:** NRAS, metformin, trametinib, combination therapy

## Abstract

Attempts to directly block the mutant neuroblastoma rat sarcoma oncogene (NRAS) protein, a driving mutation in many cancer types, have been unsuccessful. Current treatments focus on inhibition of different components of NRAS' two main downstream cascades: PI3K/AKT/mTOR and MAPK. Here we test a novel dual therapy combination of metformin and trametinib on a panel of 16 NRAS mutant cell lines, including melanoma cells, melanoma cells with acquired trametinib resistance, lung cancer and neuroblastoma cells. We show that both of the main downstream cascades of NRAS can be blocked by this combination: metformin indirectly inhibits the PI3K/AKT/mTOR pathway and trametinib directly impedes the MAPK pathway. This dual therapy synergistically reduced cell viability *in vitro* and xenograft tumor growth in vivo. We conclude that metformin and trametinib combinations are effective in preclinical models and may be a possible option for treatment of NRAS mutant cancers.

## INTRODUCTION

Mutations in the neuroblastoma rat sarcoma viral oncogene (*NRAS*) play an important role in cancer. *NRAS* mutations are found in 15-20% of malignant melanomas, but also in several other cancer types [1-5]. These point mutations usually affect codons 12, 13 (Exon I) and 61 (Exon II) of the *NRAS* gene. The mutant NRAS protein constitutively activates downstream signaling cascades such as the MAPK, PI3K/AKT/mTOR and Ral pathways, resulting in uncontrolled cell proliferation and tumor growth [1,6,7]. Attempts to directly inhibit mutant NRAS have been unsuccessful so far. Treatment approaches use small molecule inhibitors which interfere with NRAS downstream pathways [8-10]. Mitogen-activated protein kinase kinase (MEK) inhibitors block signaling through the MAPK pathway and NRAS mutated tumors are associated with MEK inhibitor efficacy [11]. The MEK inhibitor trametinib has shown clinical efficacy in patients with NRAS mutant cancers [9].

Given the activation and the crosstalk of MAPK and PI3K/AKT/mTOR pathways in *NRAS* mutant tumors, and the development of resistance to monotherapies, there is a strong rationale for a dual pathway inhibition. Various inhibitor combinations have shown preclinical efficacy and are currently being evaluated in trials [5,9,10,12-14]. So far, the outcomes are only modest and the use of such combinations is partly limited by serious adverse events [15-17].

On the other hand, colleagues report encouraging preliminary results on trials testing metformin alone or in combination with vemurafenib in *BRAF^V600^* mutant melanoma (NCT01840007; NCT01638676) [18]. Metformin is a biguanide which has been used as an oral anti-diabetic drug for decades and has a well-known safety profile. In addition to its effects on glucose metabolism, it ultimately inhibits mTOR in cancer cells and leads to growth arrest and apoptosis [19,20]. Retrospective studies report a decrease in cancer risk and lower cancer mortality in diabetic patients treated with metformin [21-24]. A recent meta-analysis includes 65,540 cancer cases in diabetic patients and shows that the cancer incidence and mortality in those taking metformin was reduced. However, the authors state that the results varied significantly across studies and that prospective studies in non-diabetic patients are needed to understand metformin's effect on cancer [25].

Consequently, we explore if metformin could be a potential partner of trametinib for the treatment of *NRAS* mutant tumors. The combination would lead to a desirable dual pathway inhibition. We use a large panel of *NRAS* mutant melanoma, neuroblastoma and lung cancer cell lines. We show that the combination of metformin and trametinib has a synergistic effect in NRAS mutant tumors and reduces tumor size in a xenograft model. In addition, we investigate the effect of metformin on two *NRAS* mutant melanoma cell lines with acquired resistance to trametinib.

## RESULTS

### Effects of metformin and trametinib on NRAS mutant melanoma cells

**Figure 1:**
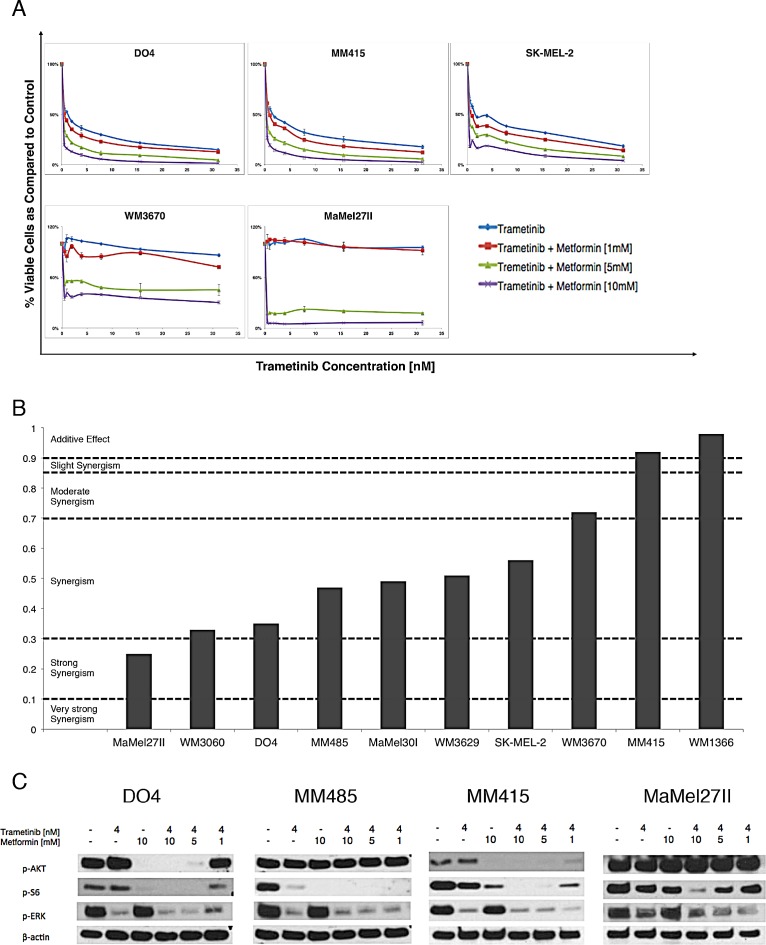
Metformin and trametinib have synergistic effects on cell viability in NRAS mutant melanoma cells (A) Representative dose response curves for trametinib and its combination with metformin in different ratios. The addition of metformin to trametinib leads to a downward shift of the dose response curves in all cell lines tested (n=3, incubation 72hrs, error bars represent SD). (B) Combination index (CI) values for metformin and trametinib. The CI values were calculated using the CalcuSyn software according to the recommendations of Chou-Talalay. The drugs show synergism in all cell lines except MM415 and WM1366 where they have an additive effect [26] (C) Immunoblot analyses for downstream effector proteins of the MAPK and PI3K/AKT/mTOR signaling pathways. Cells were treated with metformin, trametinib or their combinations (incubation 4 hrs.). Dual pathway inhibition is achieved by combining metformin and trametinib, as evidenced by the decrease of p-ERK and p-S6.

To evaluate the response of *NRAS* mutant melanoma to metformin and trametinib, we first performed CTG-cell viability assays. We incubated a panel of 10 cell lines with previously characterized mutations [12] with the respective drugs and their combinations. The cell lines, their mutations and GI50 values (concentrations of drugs resulting in 50% decrease in cell viability relative to controls) for the single agent drugs are reported in Table 1. The GI50 values in treatment-naïve melanoma cells ranged from 3.39 to 14.45mM with an average value of 7.11mM for metformin and from 0.67 to >100nM for trametinib. All cell lines showed more cell viability decrease when the drugs were combined (Figure 1A).

**Table 1:** Growth inhibitory effects of metformin and trametinib in NRAS mutant cancer cell lines The table displays concentrations of drugs resulting in 50% decrease of cell viability relative to untreated controls (GI50). Drug concentrations ranged from 0.1-20mM for metformin and 0.2-30nM for trametinib

Cell Line	Tissue	NRAS mutation	GI50 value trametinib [nM]	GI50 value metformin [mM]
DO4	Melanoma	Q61L	0.7	5.2
MM415	Melanoma	Q61L	1.3	7.15
WM1366	Melanoma	Q61L	55.9	4.36
SK-MEL-2	Melanoma	Q61K	0.67	5.32
WM3060	Melanoma	Q61K	1.94	9.96
MM485	Melanoma	Q61R	1.2	14.45
MaMel30I	Melanoma	G13D	15.66	5.52
MaMel27II	Melanoma	G12D	>100	6.4
WM3629	Melanoma	G12D	9.92	3.39
WM3670	Melanoma	G12D	52.0	9.3
DO4-RM	Melanoma	Q61L	23.3	10.16^a^
MM415-RM	Melanoma	Q61L	>100	3.22^a^
SW-1271	Lung carcinoma	Q61R	26.8	29.9
NCI-H2347	Lung carcinoma	Q61R	82.0	6.79
SK-N-AS	Neuroblastoma	Q61K	5.82	3.52
CHP-212	Neuroblastoma	Q61K	0.26	1.11

a Experiments in DO4-RM and MM415-RM cells with acquired trametinib resistance were performed in the presence of 5nM and 55nM trametinib, respectively.

To analyze whether the combination leads to inhibition of cell proliferation or cell death, we studied the induction of apoptosis or necrosis by staining the cells with Annexin V/Propidium Iodide assay followed by flow cytometry (Supplementary Figure S1). These assays also served as a control to the CTG assay, which measures total ATP levels in cells and might report incorrect measurements of cell viability, especially when mitochondrial complex I inhibitors like metformin are used. The apoptosis assays showed that the combination leads to cell death and they confirmed the synergistic results of the CTG assays.

To quantify the effect of the combination, we used Calcusyn software to calculate the combination index (CI) for all melanoma cell lines. The CI values ranged from 0.25 for MaMel27II to 0.98 for WM1366 (Figure 1B). Most cell lines had a CI index between 0.3 and 0.7 indicating synergism according to the method of Chou-Talalay [26]. The combination showed an additive effect in cell lines MM415 and WM1366 who had a CI index above 0.9. No cell line showed an antagonistic effect to the combination therapy.

Next, we analyzed the effect of the drugs and their combinations on the two main NRAS downstream signaling pathways MAPK and PI3K/AKT/mTOR. Metformin showed an effect on the PI3K/AKT/mTOR pathway, where it inhibited the phosphorylation of S6 in a dose-dependent manner. In cell lines DO4 and MM415 metformin also inhibited AKT phosphorylation. Trametinib led to an inhibition of ERK phosphorylation in all cell lines tested. The combination of metformin and trametinib suppressed the phosphorylation of NRAS effector proteins ERK and S6, providing an explanation of the effects on cell viability (Figure 1C).

### Effects of metformin and trametinib on non-melanoma NRAS mutant lung cancer and neuroblastoma

**Figure 2:**
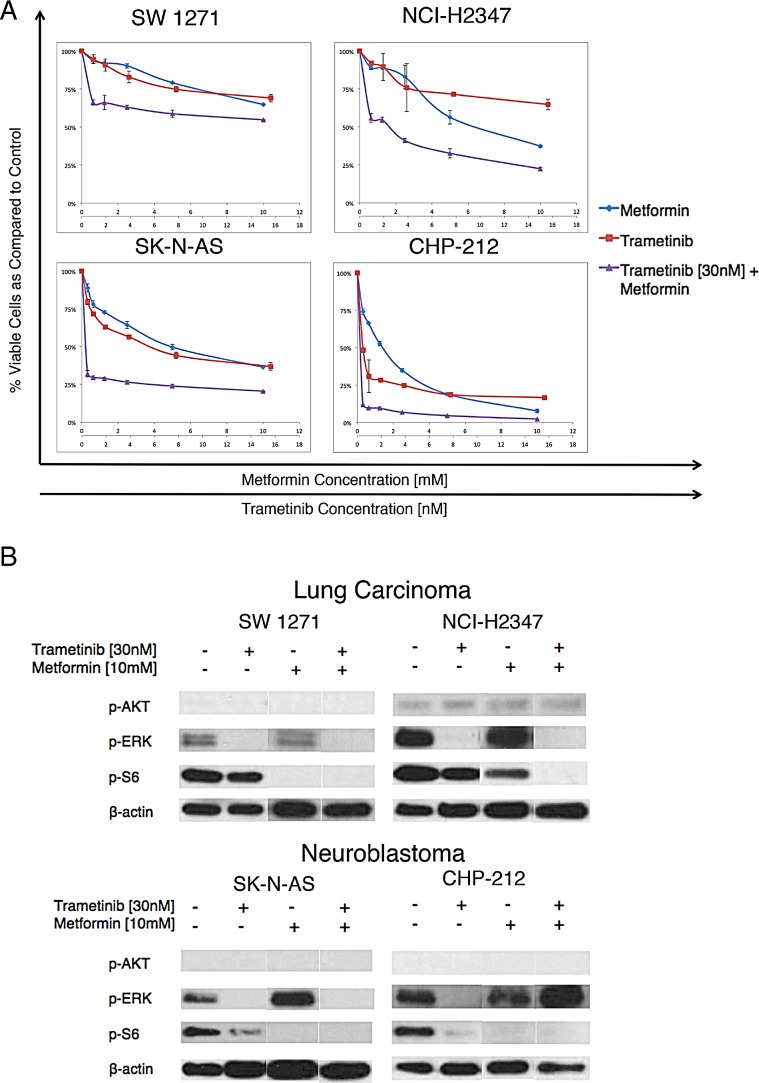
Metformin and trametinib effects on NRAS mutant lung cancer and neuroblastoma cells (A) Dose response curves for two lung carcinoma and two neuroblastoma cell lines with known activating NRAS mutations (n=3, incubation 72hrs, error bars represent SD). The combination is more effective in reducing cell viability than each of the agents alone. (B) Cropped immunoblot analyses for downstream effector proteins of the MAPK and PI3K/AKT/mTOR signaling pathways for NRAS^Q61^ mutant lung carcinoma and neuroblastoma cell lines. Dual pathway inhibition can be achieved by combining metformin and trametinib, as evidenced by the abolishment of p-ERK and p-S6.

Since NRAS shares signaling similarities across different malignancies, we hypothesized that the effects of the combination might be translated to *NRAS* mutant cancers other than melanoma [10]. For these experiments we used two neuroblastoma and two lung cancer cell lines with known *NRAS^Q61^* mutations and dependency on NRAS signaling [10]. Metformin and trametinib had comparable effects on cell viability and pathway inhibition as in *NRAS* mutant melanoma cell lines (Figure 2 A,B).

### Metformin effects on *NRAS* mutant cell lines resistant to trametinib

**Figure 3:**
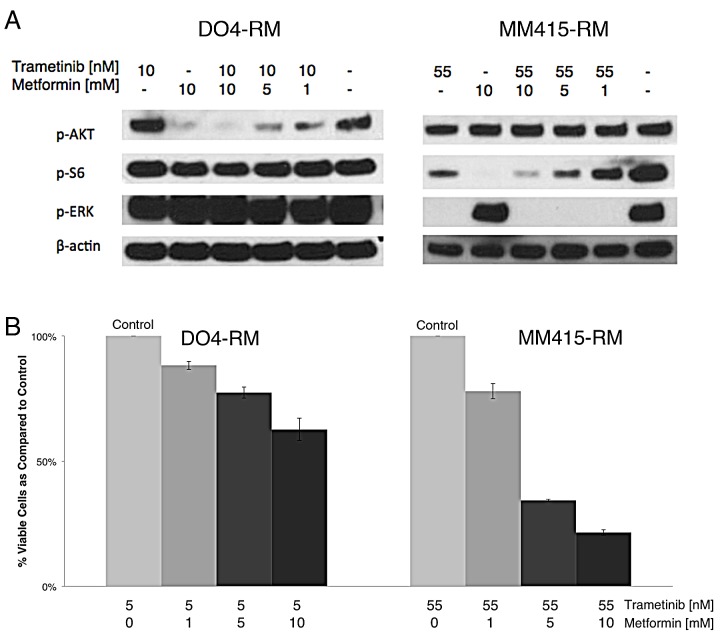
NRAS mutant melanoma cell lines with an acquired resistance to trametinib show cell viability decrease to single agent metformin (A) Immunoblot analyses of downstream effectors of NRAS mutant melanoma cells with acquired resistance to trametinib. Metformin decreases p-AKT in DO4-RM and p-S6 in MM415-RM in a dose-dependent manner. (B) Decrease of cell viability in trametinib-resistant cell lines DO4-RM and MM415-RM after treatment with metformin. Bars represent percentages of viable cells compared to controls (Incubation 72hrs, n=3, error bars represent SD).

Resistance to single agent inhibitors, such as trametinib, is a growing concern. Metformin decreased cell viability in cell lines WM3670 and MaMel27II with relatively innate trametinib resistances (Table 1, Figure 1A). Therefore, we tested metformin on trametinib-resistant cell lines DO4-RM and MM415-RM, where it decreased cell viability in both clones. The analysis of NRAS downstream effector proteins showed a dose-dependent decrease of AKT or S6 phosphorylation (Figure 3 A,B).

### Effects of metformin and trametinib on a human melanoma xenograft model

**Figure 4:**
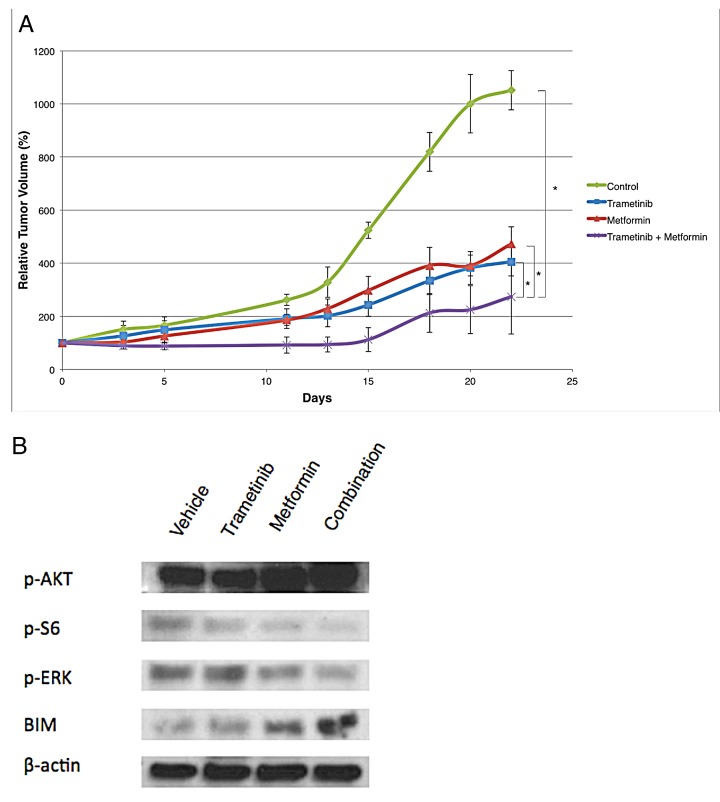
Relative tumor volume and immunoblot analyses for the respective treatment groups in a DO4 melanoma xenograft model (A) Growth curves of DO4 NRAS mutant melanoma in a xenograft model are shown. DO4 cells were implanted subcutaneously into nude mice and treatment was started when tumor volumes reached approximately 100mm^3^. Metformin and trametinib combination inhibits tumor growth more than each drug alone. The results remain statistically significant after 22 days (n>3, * p< .05, treatment start at day 0). (B) Immunoblot analyses of downstream effector proteins in DO4 xenograft tumors. The combination of metformin and trametinib leads to a stronger decrease of NRAS downstream effectors p-ERK and p-S6, and to induction of the pro-apoptotic protein BIM.

Given the promising *in vitro* results, we examined the effect of the combination in a xenograft model. Human DO4 cells were injected subcutaneously into nude mice, and gave rise to tumors in 1-2 weeks. Treatment with either metformin or trametinib decelerated tumor growth compared to vehicle treated controls. Combination therapy led to less tumor growth compared to single-drug therapy and vehicle treated controls. (Figure 4A).

The difference in relative tumor volumes was statistically significant comparing the combination group with the three other groups (vehicle, metformin only, trametinib only) on days 15 and 22 after treatment began (*p*<.05; ANOVA, Bonferroni correction). However, there was no significant difference in tumor suppression when comparing the two single treatment groups with each other (*p*=0.578, day 15; *p*=1.0, day22). Target inhibition was assessed by immunoblotting of xenograft tumor tissue. The combination treatment suppressed NRAS downstream effectors more potently than single-drug treatment. Additionally, the combination of metformin and trametinib caused an induction of the pro-apoptotic protein BIM (Figure 4B).

## DISCUSSION

Diabetes is associated with an increased risk of having certain cancers [27]. However, diabetic patients who take the blood glucose lowering metformin have lower cancer incidence, lower cancer related mortality, and lower risk of cancer development, compared to diabetics treated with other agents [18,21]. In one prospective trial metformin reduced the number of pre-cancerous lesions in the rectum, suggesting a preventive role of metformin in the development of rectal cancer [28].

The biguanide metformin is widely prescribed and has a well-known safety profile. In cancer cells, metformin interferes with the PI3K/AKT/mTOR pathway, and indirectly inhibits mTOR. It leads to cell cycle arrest, increased apoptosis and autophagy [18-20,29]. This makes metformin an attractive candidate for the treatment of *NRAS* mutant cancer, where the constitutively active NRAS signals through the PI3K/AKT/mTOR pathway.

This study evaluates the effects of the combination of metformin and the MEK inhibitor trametinib in *NRAS* mutant cancer. We focus our efforts on *NRAS* mutant melanoma, but also show that the same principles apply in other cancers with known oncogenic NRAS mutations. NRAS mutations are rare in lung cancer and neuroblastoma. However, the high prevalence of these tumors makes their *NRAS* mutant form clinically very relevant [10].

Several studies report inhibitory effects in different cancer types using AMPK activators like metformin, AICAR or phenformin, alone or in combination with other drugs such as erolotinib or dasatinib [30-38]. Whether *BRAF^V600^* mutant and *NRAS* mutant melanoma cells have differing sensitivities to metformin is a matter of debate. While some authors show that the induction of autophagy and apoptosis is independent of the *BRAF* or *NRAS* mutation status of cells, others report that metformin increases tumor growth of *BRAF* mutated melanoma xenografts by up-regulation of VEGF-A and induction of angiogenesis [30-32]. In our hands, metformin inhibits cell growth and induces apoptosis in *NRAS* mutant melanoma, neuroblastoma and lung cancer with GI50 values ranging between 1.11mM and 29.9mM (Table 1, Figure 1). Our results are supported by other studies, strengthening the rationale for metformin use in *NRAS* mutant cancers [30-32,39]. As previously shown, all NRAS mutant cells used in this study depend upon the NRAS mutation. Still, variations to what extent the typical downstream cascades of mutant NRAS account for survival and proliferation exist. This might, at least in part, explain the observed differences in sensitivity to the inhibitory agents used in this study.

Mutant *NRAS* constitutively signals through the MAPK and PI3K/AKT/mTOR pathways, among others. Mounting evidence suggests that dual inhibition of both pathways may lead to better results in *NRAS* mutant cancer [10,12,14]. *NRAS* mutations are associated with MEK inhibitor efficacy and first clinical results with MEK inhibitors show a response rate of 20% in patients with advanced *NRAS* mutant melanoma [11,40]. Here, we show that metformin and trametinib synergistically decrease cell viability in a large survey of *NRAS* mutant melanoma, lung cancer and neuroblastoma cell lines, including two cell lines with acquired trametinib resistance. The finding of synergism in different cancer types with activating *NRAS* mutations is not surprising, because *NRAS* mutant melanoma, lung cancer and neuroblastoma all seem to depend on NRAS downstream signaling through MAPK and PI3K/AKT/mTOR [3,10]. Trametinib inhibits the MAPK pathway while metformin inhibits the PI3K/AKT/mTOR pathway. The combination of the two produces a synergistic effect because they lead to dual pathway inhibition of NRAS's important signaling pathways. Our immunoblots verified the dual pathway inhibition by the combination, and showed a reduction in NRAS downstream cascade effector proteins p-AKT, p-S6, and p-ERK.

Resistance to targeted inhibitors is a growing concern in the field of oncology, and multiple mechanisms of such resistance are known [41-43]. We show the effect of metformin on cells with an acquired resistance to trametinib and hypothesize that its addition to trametinib might delay or reverse the development of resistance. Blocking targets in the PI3K/AKT/mTOR pathway by different drugs can reverse resistance to inhibitors of MAPK signaling cascade components, as shown by other authors in melanoma [44].

Certain questions should be addressed before metformin is used in combination therapy on patients. Metformin doses used in *in vitro* studies are higher than serum levels observed in diabetic patients (1 to 20umol/L) [45]. Yet, retrospective epidemiologic studies and meta-analyses still showcase the anti-tumor effects of metformin [22-25,46]. A possible explanation of the *in vivo* effect of the drug is the accumulation of positively charged metformin in certain tissues and within the mitochondria. This can lead to local concentrations >1000 fold higher than the serum levels [47-49]. Also, the mechanism of metformin's *in vivo* effects has not been fully understood. Beyond the blockage of the PI3K/AKT/mTOR signaling pathway, metformin could interfere with insulin signaling and lower serum glucose [27,50,51]. Here, immunoblots of xenograft tumors showed some blockage of NRAS downstream pathways when metformin was used. Combination therapy on mouse xenograft tumors showed tumor volumes significantly lower than those of the control and single treatment groups for the entirety of treatment, but we could not abolish tumor growth completely with either treatment.

Further studies are needed to elucidate the *in vivo* action of metformin, but our study provides evidence that a combination of metformin and trametinib may be a possible option for treatment of *NRAS* mutant cancers.

## METHODS

### Cell lines, cell culture

Human *NRAS* mutant melanoma cell lines DO4, MM415, MM485, SK-MEL-2, MaMel30I and MaMel27II were a generous gift from Boris Bastian at the University of California, San Francisco (UCSF); cell lines WM1366 (Cat N. WC00078), WM3629 (Cat N. WC00117), WM3670 (Cat N. WC00119) and WM3060 (Cat N. WC00126) were obtained from Coriell Institute (Wistar Institute, Philadelphia, PA, USA). *NRAS* mutant lung carcinoma cell lines SW1271, NCI-H2347 and neuroblastoma cell lines SK-N-AS and CHP212 were purchased from American Type Culture Collection (Oct/2013). Cell lines were not authenticated by short tandem repeat DNA profiling after purchase, but were regularly tested for NRAS mutation status.

Cell lines DO4, MM415, MM485, SK-MEL-2, MaMel30I, MaMel27II, SW1271, NCI-H2347, SK-N-AS and CHP212 were cultured in RPMI-1640 media supplemented with 10% (vol/vol) heat inactivated fetal bovine serum (FBS); cell lines WM1366, WM3629, WM3670 and WM3060 were cultured in MCDB153 media supplemented with 20% (vol/vol) Leibovitz's L-15 media, 2% (vol/vol) FBS, and 1.68 mM CaCl2. All cell lines were incubated at 37 °C under 5% CO2.

### Establishment of trametinib resistant cell lines DO4-RM and MM415-RM

We treated cell lines D04 and MM415 with trametinib dosages corresponding to their respective GI50 values and increased trametinib concentrations over a period of approximately 6 months. Surviving clones were selected for and further propagated. The established resistant cell lines were termed DO4-RM and MM415-RM were maintained in RPMI 1640 media supplemented with 10% (vol/vol) FBS and 5nM or 55nM trametinib, respectively (Supplementary Figure S2).

### Drugs, cell viability assays, apoptotic assays

Trametinib and metformin were purchased from Selleck Chemicals (Houston, Texas, USA). For cell viability assays, cells were plated in 96-well plates with a density of 4000-8000 cells per well and incubated for 24 h at 37 °C with 5% C02. Then cells were incubated with increasing drug concentrations and their combinations. Cell viability was measured with the CellTiter-Glo (CTG) Luminescent Cell Viability Assay (Promega; Madison, Wisconsin, USA) according to the manufacturer's protocol. Luminescence was measured on the SynergyHT plate reader (BioTek, Vermont, USA) using Gen5 software (Version 1.11.5). For apoptotic assays, cells were plated in 12-well plates and treated with DMSO, trametinib, metformin or combinations. After 72hrs apoptosis was assessed using the Dead Cell Apoptosis Kit with Annexin V Alexa Fluor 488 & Propidium Iodide according to the manufacturer's protocol (Invitrogen; V13241) with the AccuriC6 Flow Cytometer using the CFlow software (Version 1.0.227.4).

### GI50 values, Combination index

Concentrations of drugs resulting in 50% decrease in cell viability relative to controls (GI50) as well as the combination index (CI) were calculated using CalcuSyn software (Biosoft, Cambridge, UK; Version 2.1). According to the recommendation of Chou-Talalay, a CI <0.9 indicated synergistic effects of drugs; the synergism was further refined as: slight synergism (CI=0.85-0.9), moderate synergism (CI=0.7-0.85), synergism (CI=0.3-0.7), strong synergism (CI=0.1-0.3) and very strong synergism (CI<0.1) [26].

### Western Blotting

Cells were washed with ice cold phosphate buffered saline (PBS), lysed using radio-immunoprecipitation (RIPA) buffer [150mM NaCl, 1% (vol/vol) Nonidet P-40, 0.5% (wt/vol) sodium deoxycholate, 0.1% (wt/vol) SDS] in 50mM Tris HCl (pH8.0) supplemented with protease and phosphatase inhibitors (Pierce, IL, USA; 78442). Protein concentrations were determined using the BCA Protein Assay kit (Pierce; 23225) according to the manufacturer's protocol. Proteins were separated by SDS-PAGE with 4-20% gradient gels (Bio-Rad Laboratories, CA, USA; 456-1096), transferred to an Immobilon-P PVDF membrane (Millipore, MA, USA; IPVH00010), and blocked in 5% dry milk in Tris Buffered Saline, with Tween 20 (TBST) (Sigma-Aldrich). Membranes were incubated with primary and secondary antibodies, and target proteins were detected with ECL detection reagent (Pierce; 32106). β-Actin (Sigma-Aldrich) served as a loading control. Phospho-ERK (4370), phospho-AKT (4060), phospho-S6 (4857), Bim (2933) antibodies were obtained from Cell Signaling Technology (MA, USA).

### Tumor xenografts in nude mice

Six-week-old female CrTac:NCr-*Foxn1^nu^* mice (Taconic Farms, USA; weight 20-25g) were injected subcutaneously with 6 × 10^6^ DO4 cells re-suspended in 200ul Matrigel (BD Biosciences, USA). Treatment was started when tumors reached approximately 100mm^3^. Mice were treated once daily, five days per week, with either oral vehicle (methylcellulose 0.5%, Tween80 0.2%; oral gavage), trametinib (2mg/kg/day, oral gavage), D-PBS (200ul; intraperitoneal injection) or metformin (200mg/kg/day; intraperitoneal injection). The change in tumor size was measured with calipers every 2-4 days, and the tumor volume was calculated using the formula V=π x a x b x c x 4/3. After 22 days of treatment mice were euthanized. Tumors were collected, pulverized in liquid nitrogen, lysed and subjected to western blotting as described above. All mouse studies were approved by the UCSF Institutional Animal Care and Use Committee (Protocol # AN086990-03A).

## SUPPLEMENTARY MATERIAL AND FIGURES


