# Supplementary material for: Metformin and trametinib have synergistic effects on cell viability and tumor growth in NRAS mutant cancer
Source: Oncotarget. 2014 Nov 25;6(2):969–78. doi: 10.18632/oncotarget.2824 (PMC4359268; doi:10.18632/oncotarget.2824)
Supplement: Supplementary file 1 [file oncotarget-06-969-s001.pdf]

Metformin and trametinib have synergistic effects on cell viability and tumor growth in *NRAS* mutant cancer

Supplementary Material

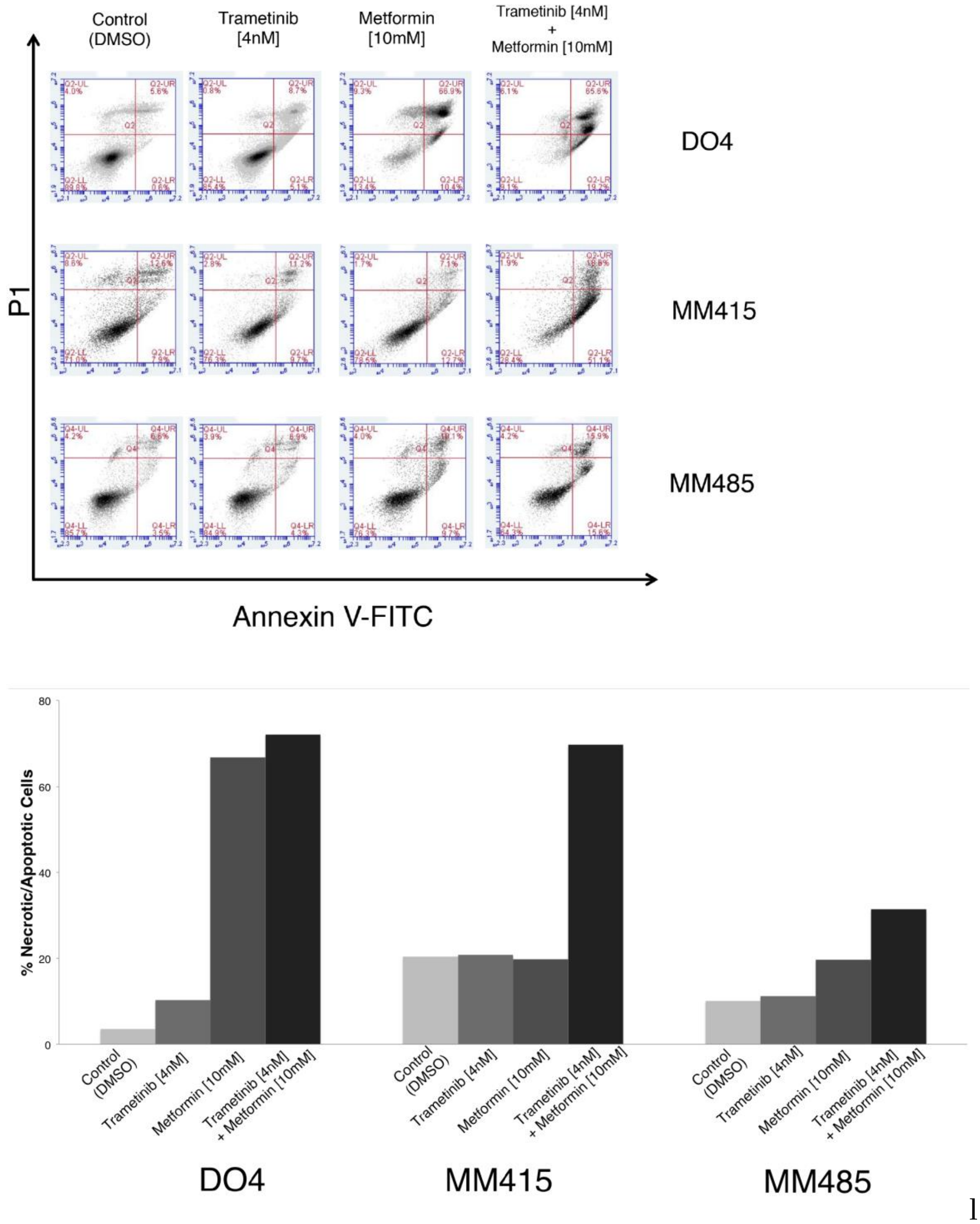

Supplementary Figure S1: Metformin and trametinib have synergistic effects on cell viability in NRAS mutant melanoma cells. Representative flow cytometry dot blots from cells treated with metformin, trametinib or their combination. The combination is more effective in inducing cell death than single agents. Bars represent the relative number of apoptotic/necrotic cells compared to the DMSO treated controls.

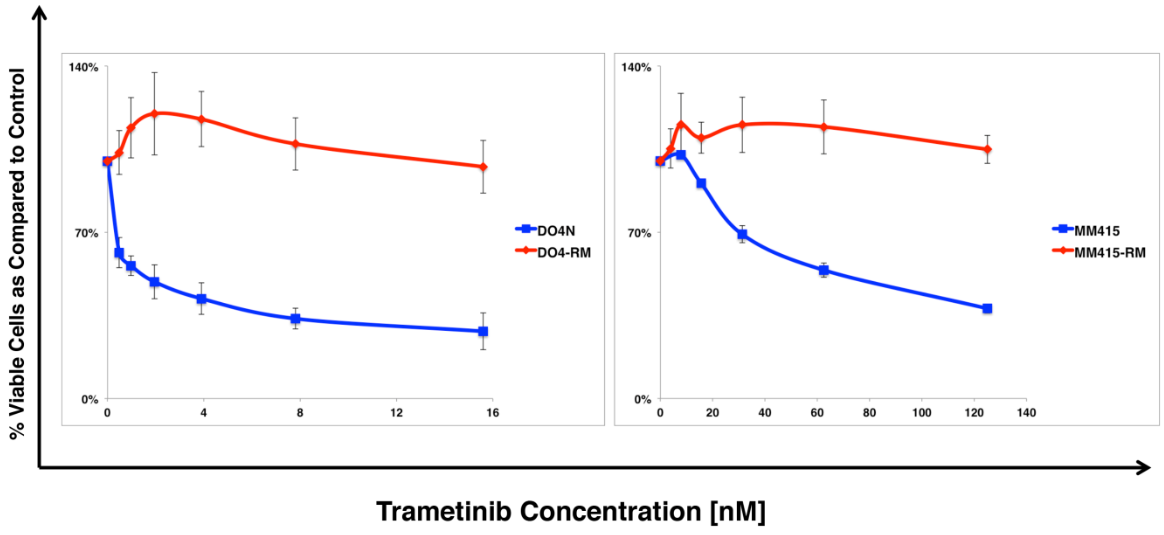

Supplementary Figure S2: Dose Response curves for two NRAS mutant melanoma cell lines and their trametinib resistant clones. Nonresistant (DO4, MM415) and established trametinib resistant clones (DO4-RM, MM415-RM) respond differently to increasing doses of trametinib (Incubation 72hrs, n=3, error bars represent SD).
